# Plasma fibroblast activation protein is decreased in acute heart failure despite cardiac tissue upregulation

**DOI:** 10.1186/s12967-024-04900-w

**Published:** 2024-02-01

**Authors:** Marta Delgado-Arija, Patricia Genovés, Lorena Pérez-Carrillo, Irene González-Torrent, Isaac Giménez-Escamilla, Luis Martínez-Dolz, Manuel Portolés, Estefanía Tarazón, Esther Roselló-Lletí

**Affiliations:** 1 Clinical and Translational Research in Cardiology Unit, Health Research Institute Hospital La Fe (IIS La Fe), Avd. Fernando Abril Martorell 106, 46026 Valencia, Spain; 2 Department of Physiology, Faculty of Medicine, Universitat de València, Avd. de Blasco Ibañez, 15, 46010 Valencia, Spain; 3 Center for Biomedical Research Network on Cardiovascular Diseases (CIBERCV), Avd. Monforte de Lemos 3-5, 28029 Madrid, Spain; 4 Heart Failure and Transplantation Unit, Cardiology Department, University and Polytechnic La Fe Hospital, Avd. Fernando Abril Martorell 106, 46026 Valencia, Spain

**Keywords:** Heart failure, FAP, Cardiac fibroblasts, Fibrosis, microRNAs, Acute heart failure

## Abstract

**Background:**

Cardiac fibroblast activation protein (FAP) has an emerging role in heart failure (HF). A paradoxical reduction in its levels in pathological conditions associated with acute processes has been observed. We aimed to identify FAP cardiac tissue expression and its relationship with the main cardiac fibrosis-related signaling pathways, and to compare plasma FAP levels in acute and chronic HF patients.

**Methods:**

Transcriptomic changes were assessed via mRNA/ncRNA-seq in left ventricle tissue from HF patients (n = 57) and controls (n = 10). Western blotting and immunohistochemistry were used to explore FAP protein levels and localization in cardiac tissue. ELISA was performed to examine plasma FAP levels in acute HF (n = 48), chronic HF (n = 15) and control samples (n = 7).

**Results:**

*FAP* overexpression in cardiac tissue is related to the expression of molecules directly involved in cardiac fibrosis, such as *POSTN, THBS4, MFAP5, COL1A2* and *COL3A1* (*P* < 0.001), and is directly and inversely related to pro- and antifibrotic microRNAs, respectively. The observed *FAP* overexpression is not reflected in plasma. Circulating FAP levels were lower in acute HF patients than in controls (*P* < 0.05), while chronic HF patients did not show significant changes. The clinical variables analyzed, such as functional class or etiology, do not affect plasma FAP concentrations.

**Conclusions:**

We determined that in HF cardiac tissue, FAP is related to the main cardiac fibrosis signaling pathways as well as to pro- and antifibrotic microRNAs. Additionally, an acute phase of HF decreases plasma FAP levels despite the upregulation observed in cardiac tissue and regardless of other clinical conditions.

**Graphical abstract:**

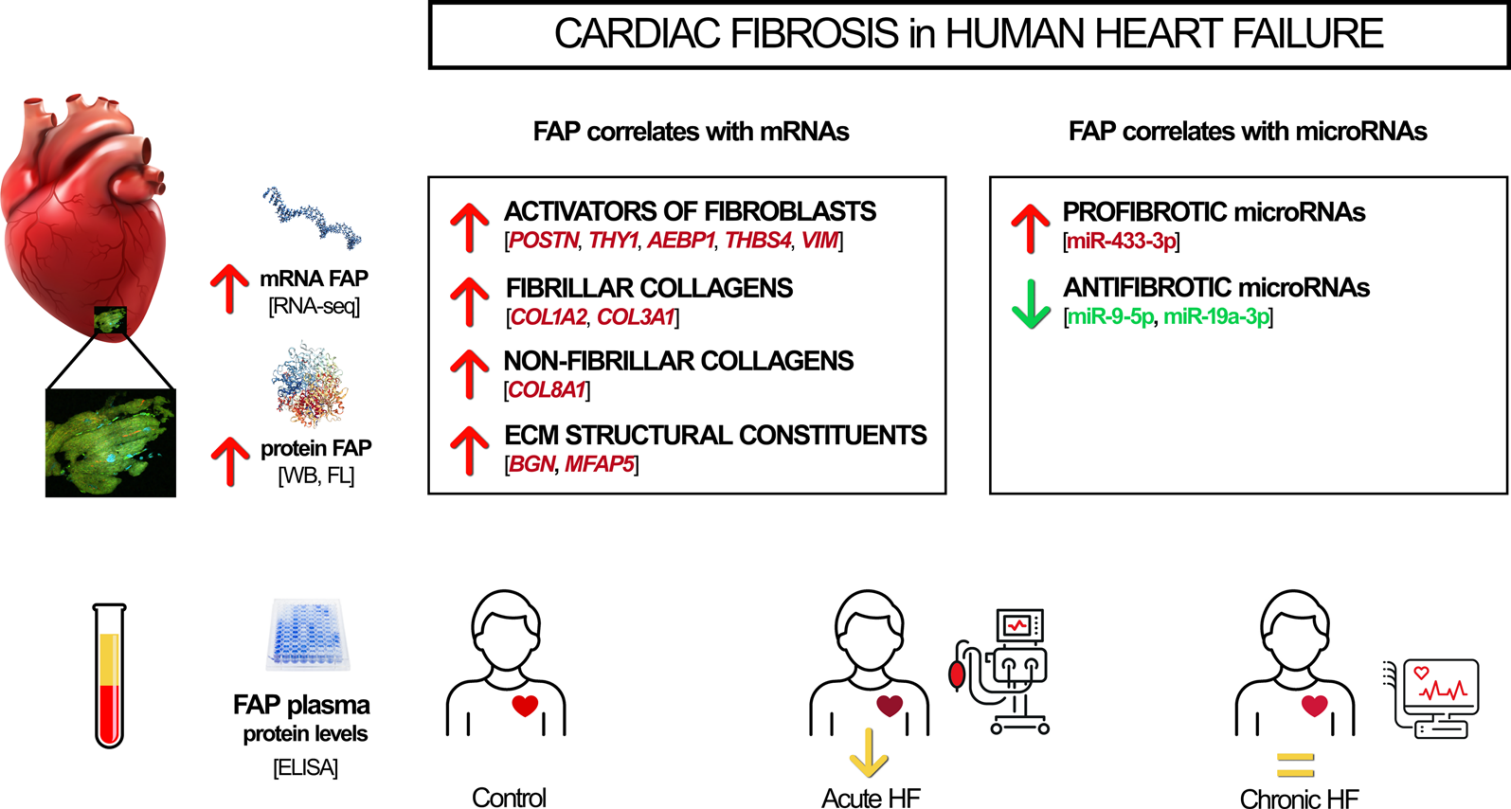

**Supplementary Information:**

The online version contains supplementary material available at 10.1186/s12967-024-04900-w.

## Introduction

Heart failure (HF) is a heterogeneous syndrome with symptoms and/or signs caused by a structural and/or functional cardiac abnormality [1]. It is one of the main causes of mortality and morbidity, being an enormous economic burden on health care systems [2]. Therefore, an urgent need exists for novel effective and targeted therapies with more precise risk stratification as well as new biomarkers for HF. This requires a deeper understanding of the underlying mechanisms driving the progression of the cardiac disease [3]. The progression of HF is accompanied by cardiac remodeling, where myocardial fibrosis plays a crucial role, and cardiac fibroblasts are the main participating cells in the development of this myocardial fibrosis [4]. Unfortunately, there is no specific therapy to reverse this myocardial fibrosis, and treatments only improve the clinical symptoms of these patients [5, 6]. Therefore, in the management of HF, it is of special interest to extend the knowledge about molecules that play a key role in cardiac fibrosis and that could be used as potential therapeutic targets. In this course of action is found the fibroblast activation protein-α (FAP), which is a type-II transmembrane serine protease expressed by activated fibroblasts almost exclusively under pathological conditions [7]. In fact, FAP is upregulated at sites of tissue remodeling, including chronic arthritis, solid tumors, and fibrotic hearts [8]. While FAP has been recognized as a potential diagnostic or therapeutic cancer target for decades [9, 10], its role in cardiac fibrosis remains unclear. Aghajanian et al., using a novel CAR-T cell therapy against FAP, observed a significant reduction in cardiac fibrosis and restoration of function after injury in mice [11]. It is known that the main cardiac fibrosis-related signaling pathways, such as the activation of cardiac fibroblasts or collagen deposition in the extracellular matrix (ECM), are altered in HF, although the association of FAP with these pathways is not fully characterized. Additionally, recent research has demonstrated that noncoding RNAs (ncRNAs), particularly microRNAs, play critical roles in the pathological development of cardiac fibrosis in HF [12]. Although certain pro- and antifibrotic microRNAs have been identified, little is known about their interactions with key fibrosis-related molecules such as FAP.

Therefore, due to the lack of knowledge on HF about the relationship of FAP with the most relevant molecules of cardiac fibrosis as well as with the expression of microRNAs involved in the fibrosis process in cardiac tissue, we analyzed its relationship with the main profibrotic genes involved in the fibroblast activation process, such as *POSTN* or *THBS4*, and with the main genes involved in the matrix remodeling process, such as *COL1A2* or *COL3A1*. Furthermore, we analyzed the expression of multiple pro- and antifibrotic microRNAs, such as miR-433-3p or miR-9-5p, and their relationship with FAP.

Recently, Sun Y et al., observed in ischemic HF that tissue levels of FAP were overexpressed while plasma FAP levels were decreased [13]. Despite having been studied, little is known about this paradoxical reduction in plasma FAP levels. Some authors suggest that this behavior of plasma FAP is reminiscent of negative acute phase proteins, which could reflect a systemic inflammatory response [13, 14]. Inflammation is accepted as an important pathophysiological factor in both acute and chronic HF predicting poor prognosis independently of left ventricular ejection fraction, although it appears to contribute in different ways to each type of HF [15]. Due to the lack of information, it is of special interest to delve into the behavior of plasma FAP levels in patients with acute HF either by rapid onset or by acute decompensation of HF and in a cohort of patients who have chronic HF. In addition, it would also be interesting to analyze whether plasma FAP levels are influenced by clinical variables such as functional class or body mass index (BMI), among other characteristics.

## Methods

### Source of samples

Left ventricular tissue samples were obtained from the explanted hearts of 57 patients with HF (nonischemic dilated and ischemic cardiomyopathy patients) undergoing cardiac transplantation and 10 control samples from non-diseased donor hearts. All control donors had normal left ventricular function (left ventricular ejection fraction > 50%) determined by echocardiography. In all cases, the cause of death was a motor vehicle or cerebrovascular accident. The control hearts were considered unsuitable for cardiac transplantation donation because of blood type or size incompatibility. Tissue samples were collected from an area proximal to the left ventricle apex, maintained in 0.9% NaCl at 4 °C for a maximum of 4.4 ± 3 h after the loss of coronary circulation and were then stored at -80 °C until RNA extraction. According to the National Transplant Organization (NTO) during 2022 in Spain, 5383 transplants were performed, of which 311 were heart transplants. Since the transplant registry started in Spain, our hospital is second in performing more heart transplants, reaching a total of 980 heart transplants. We have access to operating rooms during interventions and fully explanted hearts in all cases, so we standardized our methodology to choosing tissue samples from the same area of the left ventricle. Appropriate handling and rapid sample collection and storage led to high-quality samples (RNA ratio 260/280–2.0). Accordingly, our samples were high quality with high RNA integrity number (RIN) values (greater than or equal to 9).

Plasma samples were obtained from 66 patients who suffered HF (nonischemic dilated and ischemic cardiomyopathy patients). A total of 7 control plasma samples were obtained from subjects who presented a normal echo-Doppler study, electrocardiogram and hematologic and biochemical analyses. Blood samples were obtained at the hospital using peripheral venipuncture via a 10 mL glass vacuum extraction tube treated with 15% EDTA anticoagulant (0.12 mL) (BD Vacutainer K3E^®^; REF 368480, Becton, Dickinson and Company). The tubes were centrifuged (Eppendorf Model 5415R Centrifuge, Eppendorf Ibérica S.L.U.) at 1500 rpm for 10 min at 4 °C, and the supernatant was collected. After the venous blood was collected by venipuncture, a maximum of 2.9 ± 1.4 h passed until the samples were stored at − 80 °C. Comorbidities and other variables, such as echocardiographic data, were not available for the control groups, in accordance with the Spanish Organic Law on Data Protection 15/1999.

### Patient characteristics

Clinical history, electrocardiography, hemodynamic studies, Doppler echocardiography, and coronary angiography data were available. Table 1 shows the patient characteristics and the specific sample size of each individual study. Nonischemic dilated cardiomyopathy was diagnosed when patients had intact coronary arteries ascertained by coronary angiography and left ventricular systolic dysfunction (left ventricular ejection fraction (LVEF) < 40%) with a dilated left ventricle (left ventricular end-diastolic diameter (LVEDD) > 55 mm) assessed by echocardiography. Furthermore, none of the patients had reported a family history of the disease or showed evidence of significant primary valvular disease. Patients were diagnosed with ischemic cardiomyopathy based on the following inclusion criteria: (i) prior documented episodes of acute myocardial infarction, (ii) echocardiography showing normal contractility segments coexisting with other dyskinetic or akinetic segments, and (iii) electrocardiography showing signs of ischemia or myocardial necrosis. For the plasma study, patients were divided into two groups: (1) acute HF patients, who were hospitalized in our cardiology service because of HF, and (2) chronic HF patients, who suffered advanced HF and were included in the waiting list for heart transplantation in our medical center (La Fe University and Polytechnic Hospital, Valencia, Spain). All patients were classified according to the New York Heart Association (NYHA) functional criteria and were receiving medical treatment according to the guidelines of the European Society of Cardiology.

**Table 1:** Clinical characteristics of heart failure patients

	Cardiac tissue			Plasma		
	mRNA-Seq	ncRNA-Seq	Western Blot	ELISA		
	HF (n = 26)	HF (n = 42)	HF (n = 26)	Acute HF (n = 48)	Chronic HF (n = 15)	*P* value
Age (years)	53 ± 9	53 ± 10	50 ± 13	71 ± 14	57 ± 10	< 0.001
Gender male (%)	96	93	85	67	87	0.195
Non-ischemic dilated cardiomyopathy (%)	50	48	50	46	40	0.772
Ischemic cardiomyopathy (%)	50	52	50	54	60	
NYHA class	III–IV	III–IV	III–IV	I–IV	III–IV	0.001
BMI (kg/m^2^)	27 ± 5	26 ± 4	26 ± 5	30 ± 5	25 ± 5	0.002
Hemoglobin (mg/dL)	14 ± 3	13 ± 2	14 ± 3	12 ± 2	14 ± 2	0.002
Hematocrit (%)	40 ± 7	40 ± 6	40 ± 6	38 ± 6	43 ± 7	0.009
Total cholesterol (mg/dL)	155 ± 39	161 ± 45	154 ± 52	129 ± 33	116 ± 29	0.207
Prior hypertension (%)	25	32	36	82	67	0.283
Prior smoking (%)	71	72	64	50	40	0.561
Diabetes mellitus (%)	29	29	26	46	20	0.129
Hypercholesterolemia (%)	13	19	22	60	53	0.765
LVEF (%)	21 ± 8	21 ± 7	24 ± 8	30 ± 13	21 ± 10	0.035
LVESD (mm)	66 ± 12	61 ± 12	61 ± 12	44 ± 13	59 ± 11	0.001
LVEDD (mm)	74 ± 11	69 ± 12	69 ± 12	56 ± 10	68 ± 10	< 0.001

*HF Heart failure*, *NYHA* New York Heart Association, *BMI* body mass index, *LVEF* left ventricular ejection fraction, *LVESD* left ventricular end-systolic diameter, *LVEDD* left ventricular end-diastolic diameter

*P* value is the comparison between acute HF and chronic HF in plasma samples

### RNA extraction and integrity, mRNA-Seq, and ncRNA-Seq analysis

For mRNA-Seq, 36 samples were examined (HF, n = 26; and control, n = 10), and for ncRNA-Seq, a total of 50 samples were analyzed (HF, n = 42; and control, n = 8). RNA extraction, determination of purity and integrity of RNA samples, mRNA-Seq, and ncRNA-Seq analysis were performed as previously described by Pérez-Carrillo et al. and Gil-Cayuela et al. [16, 17]. Briefly, TRIzol^®^ agent was used to homogenize tissue samples in a TissueLysser LT (Qiagen). RNA extractions were performed using a PureLink™ Kit (Ambion Life Technologies) for mRNA-Seq and the Quik-RNATM miniprep plus kit (Zymo Research) for ncRNA-Seq, according to the manufacturer’s instructions. The cDNA libraries were obtained following Illumina’s recommendations. Sequencing was performed using the SOLiD 5500XL platform for mRNA and the Illumina HiSeq 2500 sequencer for ncRNA.

### Western blot

For Western blotting, 34 samples were analyzed (acute HF, n = 4; chronic HF, n = 22; and control, n = 8). Methods used for homogenization of samples, protein determination, polyacrylamide gel electrophoresis and Western blot analysis were performed as previously described by Cortés et al. [18]. Specifically, we used Bis–Tris electrophoresis on 4–12% polyacrylamide gels under reducing conditions. After electrophoresis, proteins were transferred to a polyvinylidene difluoride membrane (PVDF) using the iBlot Dry Blotting System (Invitrogen Ltd.), blocked at 4 °C overnight with 1% BSA in Tris buffer solution containing 0.05% Tween 20 and incubated for 2 h with the primary antibody in the same buffer. The primary detection antibodies used were anti-FAP rabbit monoclonal antibody (1:500 dilution, ab207178, Abcam) and anti-GAPDH mouse monoclonal antibody (1:1000 dilution, ab8245, Abcam) as a loading control. The bands were visualized using an acid phosphatase-conjugated secondary antibody and Sigma*fast*™ BCIP^®^/NBT (B5655-25TAB, Sigma‒Aldrich) substrate system. Finally, the bands were digitalized using an image analyzer (DNR Bio-Imagining Systems) and quantified with GelQuant Pro (v. 12.2) program.

### ELISA

FAP protein levels were measured in 70 plasma samples from patients diagnosed with acute HF (n = 48) and chronic HF (n = 15) and from healthy controls (n = 7). Assessment of FAP levels in plasma was determined via specific sandwich enzyme-linked immunosorbent assays according to the manufacturer´s instructions (FAP Human ELISA Kit, ab193701, Abcam). The FAP test has a limit of detection of 12 pg/ml, and the intra- and interassay coefficients of variation were < 10% and < 12%, respectively. No significant cross-reactivity or interference between FAP and its analogs was observed. The test was quantified at 450 nm in a dual-wavelength microplate reader (Sunrise, Tecan) using Magellan version 2.5 software (Tecan).

### Immunofluorescence staining for localization of FAP in human cardiac tissue

For the immunofluorescence assay, human heart samples previously fixed with 0.5% glutaraldehyde and 5% paraformaldehyde were used. These samples were later placed in CryoBloc (070130, Diapath) and stored at − 80 °C. The cryostat was used to obtain two sections of 7 µm thick from each sample, and they were deposited on a slide. These sections were fixed with 4% paraformaldehyde and embedded for 30 min in 10 mM sodium citrate buffer + 0.05% Tween (pH 6) at 80 °C. Then, they were incubated for 1 h with a blocking solution (2% BSA in PBS buffer with Triton X-100 0.1%). To assess the localization of activated fibroblasts, sections were double stained with primary antibody for fibroblast activation protein (1:50 dilution, ab207178, Abcam) and primary antibody for cardiac troponin T (1:50 dilution, MA5-12960, Invitrogen). As secondary antibodies, Alexa Fluor 488 (1:400 dilution, ab150077, Abcam) and Alexa Fluor 594 (1:200 dilution, A-21201, Invitrogen) were used. The coverslips were mounted using mounting medium with DAPI (ab104139, Abcam). Digital images were captured on a Nikon H550S microscope.

### Statistics

Data are expressed as the mean value ± standard deviation for continuous variables and as a percentage for discrete variables. In the text the data of each molecule is shown as the fold change (FC), conversely in the figures we show the relative expression (in arbitrary units) extracted from the RNA-seq analysis to provide an idea of the average expression of each of the molecules. In the case of FAP protein values measured by western blot, they are expressed in relative values with respect to the control group (FC). The control values have been set to 100. The Kolmogorov–Smirnov test was used to analyze the distribution of the variables (clinical characteristics and molecules analyzed). Student’s *t* test was used to compare continuous variables with a normal distribution, while the nonparametric Mann–Whitney U test was used for data that were nonnormally distributed, and Fisher’s exact test was used for discrete variables. Pearson’s correlation coefficient was calculated to determine the statistical relationship between variables that were normally distributed, while Spearman’s correlation coefficient was used for data with a nonnormal distribution.

The relativity, sensitivity, specificity, and predictive value of FAP plasma levels for the presence of acute HF were assessed by the construction of receiver operating characteristic (ROC) curves. To determine whether FAP was an independent predictor for acute HF, binary logistic regression analyses were performed. Discrimination was assessed through the C statistic. Furthermore, for the ncRNA-Seq studies, differential expression analysis between conditions was assessed using the DESeq2 method [19] (version 3.4). We considered differentially expressed ncRNAs those with a *P* value (P adj) corrected by FDR ≤ 0.05 to avoid identification of false-positives across the differential expression data [20]. HTSeq software, version 0.5.4p323, was used to determine gene expression levels, and the gene predictions were estimated using the cufflinks method [21]. With this approach, multimapped reads were eliminated, and only unique reads were taken into account when estimating gene expression. The differential mRNA expression analysis between conditions was performed using the edgeR technique, version 3.2.4 [22]. This method uses various normalization procedures based on in-depth global samples, CG composition, and gene length. This method uses a Poisson model to estimate the variance of the RNA-seq data for differential expression during the differential expression process. Significance was assumed as *p* < 0.05. All statistical analyses were performed using SPSS software v. 20 for Windows (version 20.0; IBM SPSS Inc.) and R commander program (version R-4.3.1).

## Results

### Clinical characteristics of patients

The study patient populations for each assay are described in Table 1 and in methods. Briefly, we analyzed myocardial human heart samples from patients undergoing transplantation, while the control samples were obtained from non-diseased donor hearts. In addition, plasma samples from patients with acute and chronic HF and healthy controls were also analyzed.

### Alteration of FAP expression in HF patients. Relationship with genes related to cardiac fibrosis

**Fig. 1:**
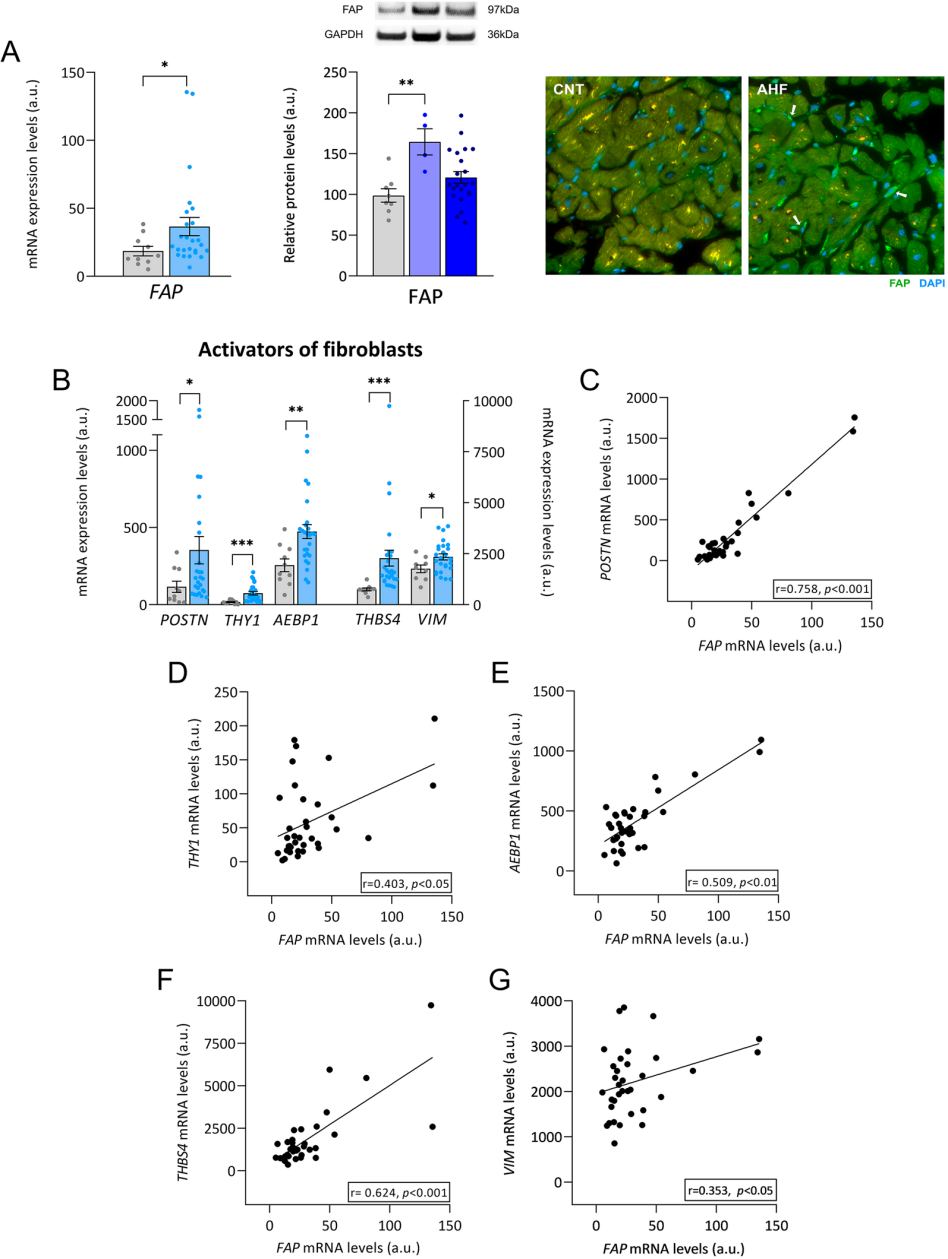
Cardiac *FAP* is upregulated and related to other activators of fibroblasts in HF. **A** Relative mRNA expression in control subjects (n = 10) and patients with HF (n = 26); and relative protein levels of FAP in control subjects (n = 8) and patients with AHF (n = 4) and chronic HF (n = 22) which are expressed in relative values with respect to the control group (FC). The control protein values have been set to 100. Immunohistochemistry of FAP (green) and DAPI (blue) expression in CNT and AHF patient. Arrows point to FAP-labeled activated fibroblasts. The yellow/orange spot is a lipofuscin particle. **B** Relative mRNA expression levels of genes with a profibrotic function in cardiac fibrosis in control subjects (n = 10) and patients with HF (n = 26). **C–G** Correlations between *FAP* mRNA levels and profibrotic gene mRNA levels. Bars represent mean ± SEM values. Figures reflect the relative expression (in arbitrary units) extracted from the mRNA-seq analysis for both patients and controls to provide an idea of the average expression of each of the molecules. A.u., arbitrary units. Correlations were determined using Spearman’s correlation coefficient. Control subjects (gray), HF patients (light blue), acute HF (mid blue), CHF (dark blue). *p < 0.05, **p < 0.01, ***p < 0.001. *AEBP1 *ae binding protein 1, *AHF* acute heart failure, *CNT* control, *FAP* fibroblast activation protein, *HF* heart failure, *POSTN* periostin, *THBS4* thrombospondin 4, *THY1* thy1 cell surface antigen; *VIM* vimentin

We analyzed the expression of profibrotic genes that are associated with the activation of cardiac fibroblasts in samples of human cardiac tissue. For this reason, we decided to focus on the expression profile of FAP, which is a specific marker of activated cardiac fibroblasts and plays a vital role in myocardial injury and fibrosis. We found that the mRNA expression (FC = 1.98, *p* < 0.05) of *FAP* was higher in HF that in the control group (Fig. 1A). When we discriminated between acute and chronic patients at the protein levels, we observed that the patients diagnosed with acute HF showed significantly higher relative levels of FAP (FC = 1.67, *p* < 0.01) compared to the control group. Chronic HF patients maintain a statistical trend towards increasing FAP levels (FC = 1.25, *p* = 0.098) compared to the control group (Fig. 1A). We extended this discovery by showing that FAP is specifically expressed by activated cardiac fibroblasts, which are located between cardiomyocytes, in left ventricle tissue (Fig. 1A). Furthermore, we found that mRNA expression levels were altered in profibrotic genes, such as periostin (*POSTN*) (FC = 3.05, *p* < 0.05), thy-1 cell surface antigen (*THY1*) (FC = 4.33, *p* < 0.001), AE binding protein 1 (*AEBP1*) (FC = 1.85, *p* < 0.01), thrombospondin 4 (*THBS4*) (FC = 3.03, *p* < 0.001) and vimentin (*VIM*) (FC = 1.33, *p* < 0.05), whose activity is relevant in the activation of fibroblasts as well as in the advancement of fibrosis (Fig. 1B). Interestingly, all these overexpressed genes showed a direct correlation (*p* < 0.05) with *FAP* mRNA expression (Fig. 1C–G).

**Fig. 2:**
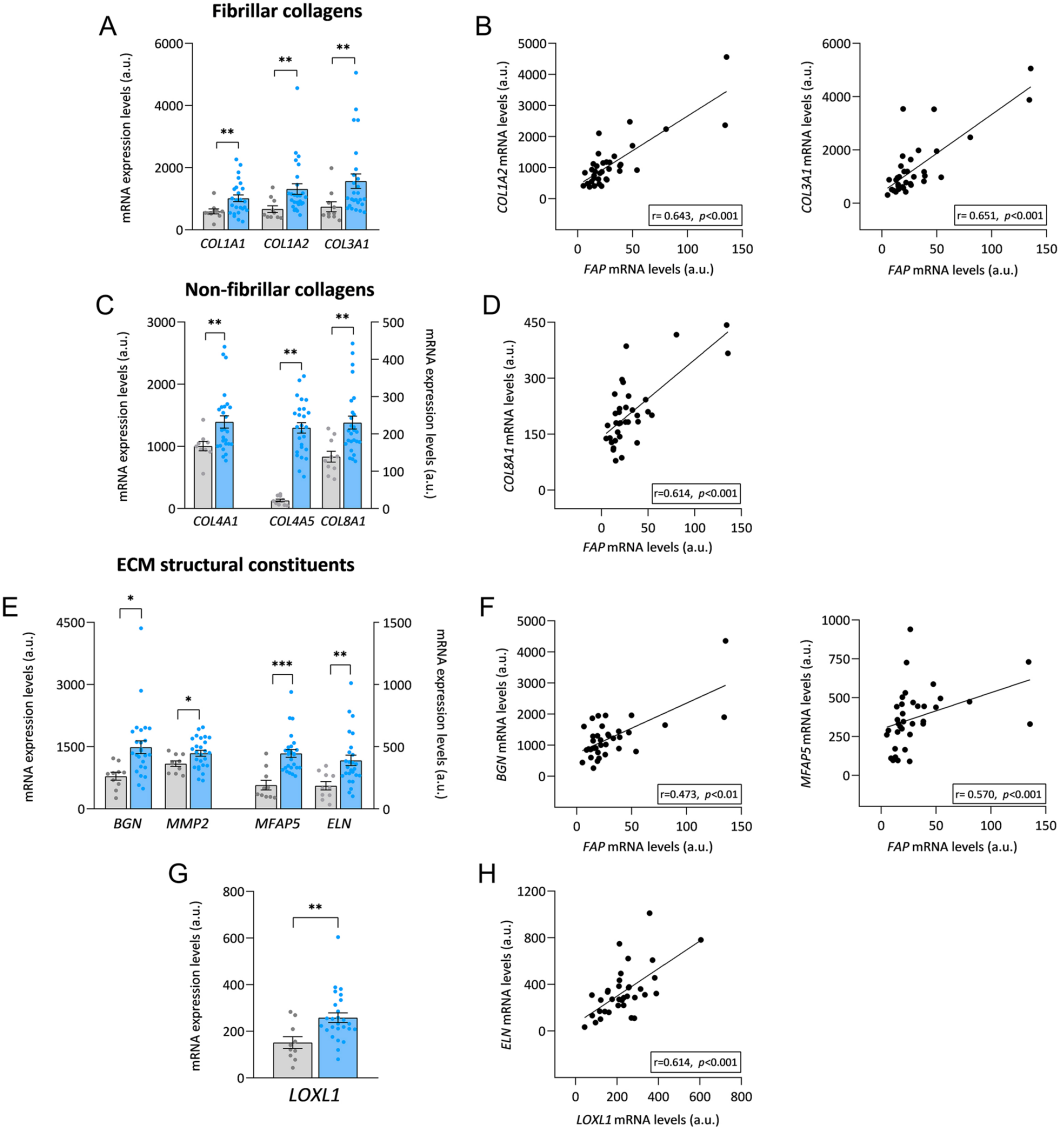
Relationship of cardiac *FAP* with genes associated with the cardiac extracellular matrix. **A** Relative mRNA expression levels of fibrillar collagens type I and type III. **B** Correlations of *FAP* mRNA levels with *COL1A2* and *COL3A1* mRNA levels. **C** Relative mRNA expression levels of nonfibrillar collagens type IV and type VIII. **D** Correlation of *FAP* mRNA levels with *COL8A1* mRNA levels. **E** Relative mRNA expression levels of ECM constituents. **F** Correlations of *FAP* mRNA levels with *MFAP5* and *BGN* mRNA levels. **G** Relative mRNA expression levels of *LOXL1*. **H** Correlation between *LOXL1* mRNA levels and *ELN* mRNA levels. Bars represent mean ± SEM values. In sections **A**–**F**, 10 control subjects and 26 patients with heart failure were analyzed. Figures reflect the relative expression (in arbitrary units) extracted from the mRNA-seq analysis for both patients and controls to provide an idea of the average expression of each of the molecules. A.u., arbitrary units. Correlations were determined using Spearman’s correlation coefficient. Control subjects (gray), heart failure patients (light blue). *p < 0.05, **p < 0.01, ***p < 0.001. *BGN* biglycan; *COL1A1*(*1A2*, *3A1*, *4A1*, *4A5*, *8A1*)- collagen type I (III, IV, VIII) α-1, α-2, α-5; *ECM* extracellular matrix, *ELN* elastin, *FAP* fibroblast activation protein, *LOXL1* lysyl oxidase like 1, *MFAP5* microfibril associated protein 5, *MMP2* matrix metallopeptidase 2

Cardiac fibrosis is also characterized by excessive collagen deposition in the ECM by activated fibroblasts. Accordingly, we found an overexpression in the most abundant proteins in the cardiac ECM, which are two types of fibrillar collagens: type I (*COL1A1* (FC = 1.72, *p* < 0.01) and *COL1A2* (FC = 1.97, *p* < 0.01) and type III (*COL3A1*) (FC = 2.10, *p* < 0.01) (Fig. 2A). They are also known for being specific fibroblast markers and for having a strong influence on the biomechanical properties of the ECM. Further evidence for the relationship between fibroblast activation and collagen deposition comes from positive correlations between *COL1A2* expression (r = 0.643, *p* < 0.001) and *COL3A1* expression (r = 0.651, *p* < 0.001) with *FAP* expression (Fig. 2B; Additional file 1: Table S1).

In addition, we observed increased expression levels in members of the nonfibrillar collagen family, which may play important regulatory roles in cardiac fibrosis. In HF patients, the mRNA expression levels of collagen IV (*COL4A1* (FC = 1.39, *p* < 0.01) and *COL4A5* (FC = 1.68, *p* < 0.01) and collagen VIII (*COL8A1* (FC = 1.66, *p* < 0.01)) were higher than those in the control group (Fig. 2C). We also found that *COL8A1* (r = 0.614, *p* < 0.001) was directly correlated with *FAP* (Fig. 2D; Additional file 1: Table S1), which maintains their possible contribution to fibrosis.

On the other hand, we examined the ECM constituent genes to assess the capacity of fibroblasts for matrix remodeling in HF patients. Compared with the control group, we observed increased expression of genes such as biglycan (*BGN*) (FC = 1.89, *p* < 0.05), matrix metallopeptidase 2 (*MMP2*) (FC = 1.23, *p* < 0.05), microfibril associated protein 5 (*MFAP5*) (FC = 2.34, *p* < 0.001) and elastin (*ELN*) (FC = 2.10, *p* < 0.01) in HF patients (Fig. 2E). In addition, we found that *BGN* (r = 0.473, *p* < 0.01) and *MFAP5* (r = 0.570, *p* < 0.001) were positively correlated with *FAP* (Fig. 2F; Additional file 1: Table S1). These results show a relationship between fibroblast activation and changes in ECM proteins in HF patients.

As we have found overexpression in collagen fiber types I and III, which are the principal characters at the collagen cross-linking process, we wanted to characterize the expression changes of the lysyl oxidase (LOX) family in HF patients because of their contribution to matrix cross-linking. We found that *LOXL1* mRNA expression was notably elevated (FC = 1.70, *p* < 0.01), which is known to be involved in elastin cross-linking (Fig. 2G). This discovery was closely matched with the *ELN* overexpression that we previously observed (Fig. 2E). In addition, we found a direct correlation between *LOXL1* and *ELN* expression (r = 0.614, *p* < 0.001) (Fig. 2H; Additional file 1: Table S1).

**Fig. 3:**
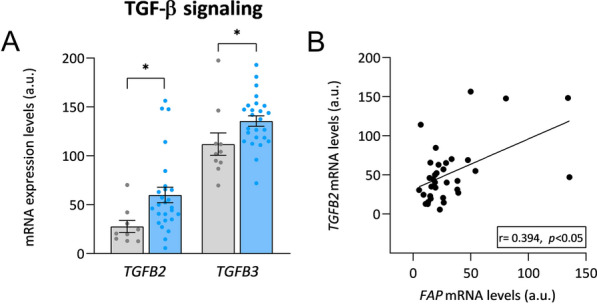
Relationship of cardiac *FAP* with members of the TGF-β signaling pathway. **A** Relative mRNA expression levels of *TGFB2* and *TGFB3* in control subjects (n = 10) and patients with heart failure (n = 26). **B** Correlation of *FAP* mRNA levels with *TGFB2* mRNA levels. Bars represent mean ± SEM values. Figures reflect the relative expression (in arbitrary units) extracted from the mRNA-seq analysis for both patients and controls to provide an idea of the average expression of each of the molecules A.u., arbitrary units. Correlation was determined using Spearman’s correlation coefficient. Control subjects (gray), heart failure patients (light blue). *p < 0.05. *TGFB* transforming growth factor beta

We also observed that the mRNA expression levels of two members of the transforming growth factor beta (TGF-β) signaling pathway for fibroblast differentiation, *TGF-β2* and *TGF-β3*, were overexpressed in HF patients (FC = 2.16, *p* < 0.05, FC = 1.21, *p* < 0.05; respectively) (Fig. 3A). Furthermore, *TGF-β2* and *FAP* mRNA expression showed a direct correlation (r = 0.394, *p* < 0.05) (Fig. 3B; Additional file 1: Table S1). We did not find any alteration in the expression of *TFG-β1* in these patients. We extended the analysis to canonical and non-canonical pathways of TGB-β signaling. The results are shown in Additional file 1: Fig. S1.

### Expression profiles of microRNAs associated with cardiac fibrosis in HF patients

**Fig. 4:**
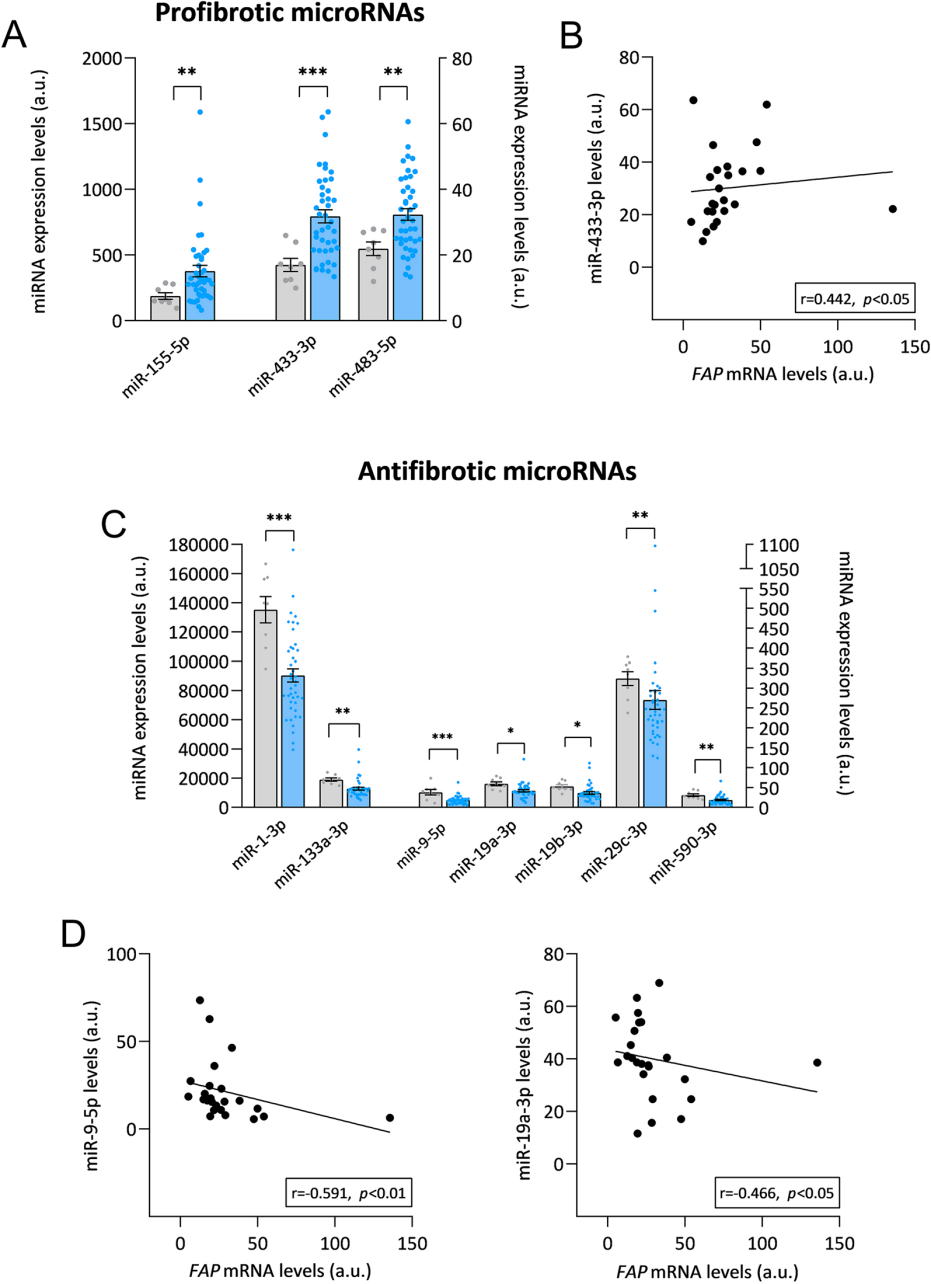
Relationship of cardiac *FAP* with pro- and antifibrotic microRNAs in HF. **A** miRNA expression levels of profibrotic microRNAs in control subjects (n = 8) and patients with heart failure (n = 42). **B** Correlation of *FAP* mRNA levels with miR-433-3p expression levels. **C** miRNA expression levels of antifibrotic microRNAs in control subjects (n = 8) and patients with heart failure (n = 42). **D** Correlations of *FAP* mRNA levels with miR-9-5p and miR-19a-3p expression levels. Bars represent mean ± SEM values. Figures reflect the relative expression (in arbitrary units) extracted from the ncRNA-seq analysis for both patients and controls to provide an idea of the average expression of each of the molecules A.u., arbitrary units. Correlations were determined using Spearman’s correlation coefficient. Control subjects (gray), HF patients (light blue). *p < 0.05, **p < 0.01, ***p < 0.001. *HF* heart failure, *miRNA* microRNA

Noncoding RNA sequencing was performed in HF patients to evaluate the expression of multiple microRNAs already known in the process of fibrosis. Indeed, we discovered microRNAs upregulated in HF patients that were related to a profibrotic function: miR-155-5p (FC = 2.01, *p* < 0.01), miR-433-3p (FC = 1.87, *p* < 0.001) and miR-483-5p (FC = 1.48, *p* < 0.01) (Fig. 4A). Subsequently, we found that miR-433-3p (r = 0.442, *p* < 0.05) showed a direct correlation with *FAP* expression (Fig. 4A; Additional file 1: Table S2). In addition, we observed a general downregulation of microRNAs related to an antifibrotic function in the cardiac tissue of HF patients: miR-1-3p (FC = − 1.50, *p* < 0.001), miR-133a-3p (FC = − 1.50, *p* < 0.01), miR-9-5p (FC = − 2.06, *p* < 0.001), miR-19a-3p (FC = − 1.42, *p* < 0.05), miR-19b-3p (FC = − 1.46, *p* < 0.05), miR-29c-3p (FC = − 1.20, *p* < 0.01) and miR-590-3p (FC = − 1.64, *p* < 0.01) (Fig. 4B). Herein, we observed that miR-9-5p (r = − 0.591, *p* < 0.01) and miR-19a-3p (r = − 0.466, *p* < 0.05), both with an antifibrotic function, were negatively correlated with *FAP* (Fig. 4B; Additional file 1: Table S2).

### Plasma FAP levels in HF patients

**Fig. 5:**
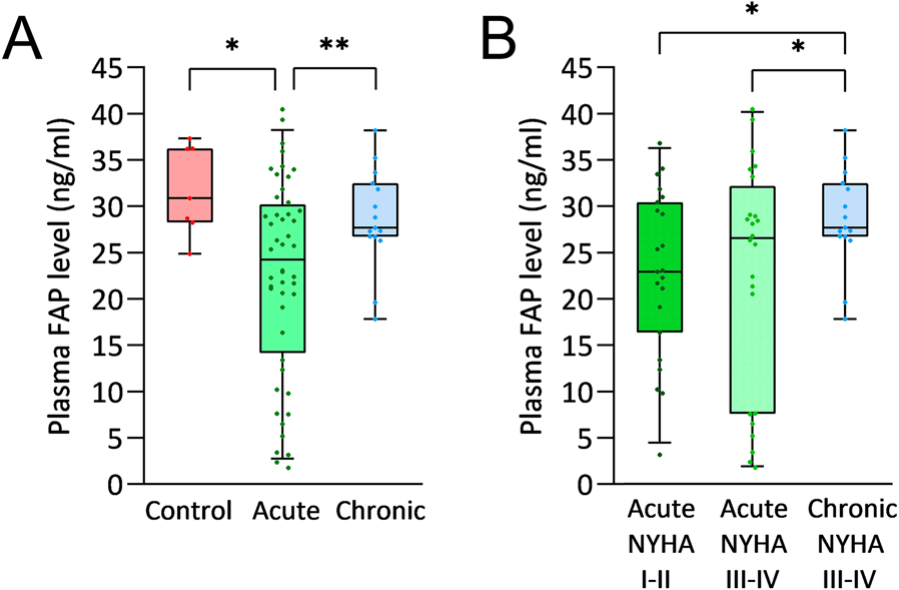
Plasma FAP levels are downregulated in acute HF patients. **A** ELISA measurement of plasma FAP levels in healthy controls (n = 7) and patients with acute HF (n = 48) and chronic HF (n = 15). **B** ELISA measurement of plasma FAP levels in acute and chronic HF patients divided by NYHA functional classification (Acute NYHA I–II, n = 23; acute NYHA III-IV, n = 24; chronic NYHA III-IV, n = 15). *p < 0.05, **p < 0.01. *FAP* fibroblast activation protein, *HF* heart failure, *NYHA* New York Heart Association

We evaluated plasma FAP levels from samples of patients diagnosed with acute and chronic HF and healthy controls. We found that the mean plasma FAP values from the acute HF patients were lower (22.58 ± 10.65 ng/ml) than those from the healthy controls (31.79 ± 4.83 ng/ml; *p* < 0.05), while there were no significant differences between plasma FAP levels of chronic HF patients (28.66 ± 5.36 ng/ml) (Fig. 5A). Furthermore, we observed that plasma FAP levels in acute HF were lower than those in chronic HF (*p* < 0.01) (Fig. 5A). In addition, we divided acute HF patients based on NYHA functional classification. We observed that there were no significant differences between acute HF patients with class I–II (22.82 ± 8.83 ng/ml) and acute HF patients with class III–IV (22.43 ± 12.53 ng/ml), but we found significant differences between acute versus chronic HF (28.66 ± 5.36 ng/ml, *p* < 0.05) with the same functional class (Fig. 5B). Our findings suggest that regardless of the functional class, the acute phase of HF decreases plasma FAP levels. We observed that the clinical variables that showed significant differences between acute and chronic HF did not affect the plasma FAP values of acute HF (Table 2). When we compared FAP levels in acute HF patients according to etiology (ischemic 22.79 ± 12.28 ng/ml and dilated cardiomyopathy 22.32 ± 8.61 ng/ml), we did not find changes.

**Table 2:** Relationships between the plasma FAP levels of acute HF and the clinical variables which showed significant differences between acute and chronic HF

Acute HF	Clinical variables	R	*P* value
Plasma FAP levels	Age (years)	− 0.141	0.341
	BMI (kg/m^2^)	0.143	0.524
	Hemoglobin (mg/dL)	0.016	0.916
	Hematocrit (%)	0.076	0.610
	LVEF (%)	− 0.143	0.400
	LVESD (mm)	− 0.079	0.666
	LVEDD (mm)	− 0.011	0.951

*BMI* body mass index, *HF* heart failure, *LVEF* left ventricular ejection fraction, *LVESD* left ventricular end-systolic diameter, *LVEDD* left ventricular end-diastolic diameter

A receiver operating characteristic curve was generated to determine the power of plasma FAP levels in distinguishing between acute HF patients and healthy controls. An area under the curve of 0.771 ± 0.077 (95% confidence interval, 0.620–0.922; *p* < 0.05) was obtained. The cutoff value of plasma FAP with 86% specificity and 96% predictive positive value was 28.18 ng/ml. Furthermore, a logistic regression analysis was performed in which plasma FAP concentration was considered an independent categorical variable (≥ 28.18 ng/ml; cutoff value selected from the receiver-operating characteristic curve for detecting acute HF), with the model adjusted by age and sex. We show that plasma FAP levels (*p* < 0.05) with an odds ratio of 9.479 were independent predictors of acute HF with a C statistic of 0.810 (95% CI 0.668–0.951, *p* < 0.01).

## Discussion

Major advances in the diagnosis and treatment of patients with HF have occurred over recent years [23]. However, despite the current treatment regimen recommended by the official guidelines, fibrosis persists in the myocardium of HF patients, worsening their prognosis [24]. Myocardial fibrosis is driven by the activation of fibroblasts [25]; in this sense, FAP has recently been proposed as a marker that exclusively identifies activated fibroblasts, allowing noninvasive quantitative measurement of early cardiac remodeling [26]. In addition, current studies have used FAP as a target to attenuate cardiac fibrosis utilizing innovative therapies such as CAR T cell technology [11, 27]. Furthermore, it has been demonstrated that the fibroblast lineage is composed of different subsets [28–30]; however, they agreed that the fibroblast subpopulations enriched in genes with a robust activation signature, including *FAP* expression, were upregulated in HF conditions [28–30].

This current study using the reliable RNA-seq technique provided new insights into the central role that FAP plays in the process of cardiac fibrosis, particularly in the activation of cardiac fibroblasts in HF patients. First, our findings suggested the activation of fibroblasts in our cohort of HF patients since we observed in their cardiac tissue samples that the mRNA and protein expression levels of FAP were upregulated. We also confirmed the presence of FAP in activated fibroblasts by immunofluorescence staining. Given that activated fibroblasts acquire greater relevance in cardiac pathological conditions, we also found that mRNA expression levels of *POSTN* and *THBS4*, which are associated with the activation of fibroblasts, were altered in HF patients. It is also significant that both genes showed a direct correlation with *FAP* mRNA expression. Interestingly, the activation of fibroblasts is considered the main mechanism of ECM remodeling in HF [31], and the relevance of alterations in the ECM in HF patients has been increasingly acknowledged [32, 33]. Collagen types I and III are the most common fibrillar collagens and are the major components of the myocardial collagen matrix [34], comprising approximately 90–95% of all cardiac collagens [35]. The relative amounts of type I vs. type III collagen fibers may be important in the regulation of mechanical properties of the myocardium and in the cross-linking process. In this study, we observed an upregulation of these fibers and correlations with *FAP* mRNA expression, suggesting a relationship between the activation of fibroblasts and collagen deposition in the ECM. Regarding the cross-linking process, we found a higher level of *LOXL1* in HF patients, which plays a crucial role as a cross-linking enzyme in fibrotic hearts. We identified several overexpressed genes in HF patients compared to the control group that encode proteins that play decisive roles in the conformation of the ECM, such as *MFAP5*, which shows a large positive correlation with *FAP*; these findings demonstrate the relationship between the activation of fibroblasts and the alteration of ECM proteins in HF patients. Additionally, we demonstrated the activation of TGF-β signaling in HF patients by identifying the upregulation of two members of the pathway, *TGFβ2* and *TGFβ3*, which has an important role in matrix remodeling and fibroblast activation [36]. It has been described that in human patients with chronic fibrotic disorders, these TGF-β signaling molecules are elevated in lung and liver tissue, while the expression of *TGFβ1* is not altered. Lower activation thresholds and more restricted expression patterns for TGFβ2 and TGFβ3 may make active TGF-β available for tissue repair in response to acute injury, which may become maladaptive in a chronic environment and lead to fibrosis [37]. In this way, Sun et al. propose selective therapeutic inhibition of TGFβ2 and TGFβ3 isoforms as a therapeutic strategy for patients with chronic fibrotic disorders [37]. Our results agree with these previous ones since we found an overexpression in *TGFβ2* and *TGFβ3*, while *TGFβ1* is not altered. Although we found altered downstream molecules of TGF-β signaling, canonical and non-canonical pathways, more studies are necessary to establish the role of FAP in the activation of TGF-β signaling. In addition to the increased secretion and deposition of structural fibrillar collagens, cardiac fibrosis is also associated with the overexpression of several nonfibrillar collagens, which can significantly affect the behavior of the ECM [38]. We detected upregulated mRNA levels of *COL4A1*, *COL4A5* and *COL8A1* in HF patients, as in previous studies [17, 39], suggesting their involvement in cardiac remodeling. Interestingly, in this study, we identified a positive correlation between *COL8A1* and *FAP* mRNA expression, sustaining their potential involvement in fibrosis.

Furthermore, with other specific RNA-seq analysis, we also detected a high number of altered microRNAs whose function has been described before in relation to the fibrosis process [40, 41]. Specifically, we observed a general increase in microRNAs whose expression is associated with profibrotic functions, such as miR-433-3p, which has been suggested as a potential target for the amelioration of cardiac fibrosis [42]. Interestingly, we discovered a direct correlation between miR-433-3p and *FAP* expression, which has not been previously described. In addition, we found underexpression of several microRNAs with an antifibrotic function, such as miR-9-5p and miR-19a-3p, whose expression was inversely correlated with the expression of *FAP*. This topic requires further investigation, given that there is no previous evidence of any relationship or regulation between these three microRNAs and FAP in cardiac fibrosis.

The origin of circulating FAP is still unknown, and previous studies have suggested that plasma FAP levels do not reflect expression levels in organ tissues [43]. However, this does not occur with genes that show expression patterns similar to those of FAP; published data indicate that these genes maintain the same trend in tissue and serum expressions [44–46]. Although it has been previously studied in other cardiovascular diseases [47, 48], to our knowledge, this is the first study to provide new information about plasma FAP levels in patients diagnosed with acute and chronic HF. In the present study, we observed the paradoxical behavior of FAP in HF since in the cardiac tissue of acute HF patients the expression levels of FAP were upregulated; however, this is not reflected at the plasma level since we observed plasma FAP was significantly lower than that in healthy controls and chronic HF patients. In addition, there were no significant differences between chronic HF and healthy controls. Therefore, our findings show that during the acute phase of HF, plasma FAP levels are decreased. Our results are in line with those of Sun Y et al., who observed in acute myocardial infarction that tissue levels of FAP were overexpressed while plasma FAP levels were decreased [13]. However, there is no explanation for this paradoxical reduction in plasma FAP levels. Uitte de Willige et al., observed in patients with coronary heart disease that the levels of soluble FAP were significantly correlated with the time between the event and study inclusion, showing decreasing plasma FAP levels with a shorter interval between the cardiovascular event and inclusion in the study [48]. Our decreased plasma FAP levels in acute HF are in line with this theory since the sample collection of patients with acute HF was performed during hospitalization after rapid onset or acute decompensation of HF, indicating a short time between the trigger of the acute event and inclusion in the study. Nevertheless, our cohort of patients with chronic HF, at the time of sample collection, had not suffered a worsening of the symptoms; instead, they suffered a chronic state of the disease. These authors also suggest that the lower plasma FAP levels in pathological conditions are reminiscent of a negative acute phase serum protein, which could reflect a systemic inflammatory response [13, 14]. Inflammation plays an important role in HF, contributing to its pathogenesis and progression [49]. In fact, elevated levels of inflammatory mediators have also been identified in acute decompensated HF; thus, there is evidence of an ongoing inflammatory response in HF [50]. Therefore, our findings about the decreased plasma FAP levels in acute HF support the theory that FAP may act as a negative acute phase serum protein in acute HF [13].

Notably, FAP expression has been studied in tissue such as a selective marker of carcinoma-associated fibroblasts and more generally, of activated fibroblasts in tissues undergoing remodelling of the extracellular matrix due to chronic inflammation, fibrosis or wound healing [48]. Also, it has been observed that FAP expression is enhanced in human fibroatheroma versus plaque-free aorta, increasing its expression upon plaque progression [8]; and the expression of FAP is increased in granulation tissue of healing wounds [47]. On the other hand, it has been seen that circulating plasma FAP decreases in acute conditions of the disease. Tillmanns et al., observed a decrease in plasma FAP levels in acute coronary syndrome, compared to healthy blood donors, and there were no significant differences between stable coronary artery disease and healthy blood donors [47, 51]. Our results are in line with those since we observed FAP overexpression in tissues in pathological conditions and a lower plasma level in the acute phase of HF compared to healthy controls. However, why plasma FAP levels decrease in these pathological conditions is still unknown. Patients with acute HF have suffered a rapid onset or an acute decompensation of HF, so at the time of sample collection these patients have acute myocardial damage, so FAP could accumulate in acute damaged myocardium, decreasing its release into the bloodstream. In fact, Tillmanns et al., already indicated that the amount of soluble FAP released from vulnerable plaques might be too small to significantly alter circulating FAP concentrations [47]. Therefore, it is possible that the amount of FAP accumulated in the injured cardiac tissue might be associated with the myocardial injury, conditioning the amount of FAP released into the bloodstream. In fact, several authors have also related circulating levels of plasma FAP to the severity of the event. In the study carried out by Tillmanns et al. in 2017, STEMI patients with increased myocardial damage exhibited a greater decrease in FAP levels after STEMI [51]. Moreover, they found a higher risk of death in coronary artery disease patients with lower plasma FAP concentrations [47]. Further studies are necessary to delve into the mechanism of FAP release in healthy and disease states.

Furthermore, some authors have related plasma FAP levels to clinical variables such as age or BMI, among others. Uitte de Willige et al., found a positive association between BMI and soluble FAP and thus higher soluble FAP levels with increasing BMI; however, they did not find an association between soluble FAP level and age or diabetes [48]. In addition, Sieweke et al., did not observe a correlation between FAP concentrations and age, and their results are in line with those of Tillmans et al. [43, 47]. In the present study in acute HF, we did not find an association between plasma FAP levels and those clinical variables that were significantly different between acute and chronic HF, such as age or BMI. Thus, we observed that plasma FAP levels decreased in acute HF regardless of the functional class since we did not find significant differences between patients with acute HF class I–II and patients with acute HF class III–IV. Furthermore, we observed significantly lower levels of plasma FAP between patients with acute HF class I–II and patients with acute HF class III–IV compared with chronic HF class III–IV patients. These findings are the first to suggest that in HF, plasma FAP levels decrease significantly during an acute phase of the disease. Further studies are needed to analyze the mechanisms underlying how in an acute phase of HF plasma FAP levels decrease, as well as whether FAP could act as a negative acute phase protein. Moreover, follow-up studies are needed to assess the variability of this molecule.

Some limitations of our study need to be emphasized. We did not measure the dynamic variations in plasma FAP levels during the development of HF. This is a study with a limited number of subjects per group that requires future studies that include large patient populations. We do not have data from chronic patients classified between class I–II by the NYHA in the collected serum samples, although we can confirm that the functional class does not influence the circulating FAP levels. Only samples from the left ventricle apex were used; therefore, these results cannot be extrapolated to other regions of the heart. However, since the samples were obtained after a heart transplantation, it has allowed the examination of a specific region of interest, something that would not have been possible from biopsied tissue.

## Conclusions

In cardiac tissue of HF patients, overexpressed FAP is related to relevant molecules of the main cardiac fibrosis signaling pathways*,* specifically to the fibroblast activation process and ECM remodeling, showing a direct correlation with profibrotic and inverse correlation with antifibrotic microRNAs. In addition, we demonstrate that FAP cardiac tissue overexpression is not reflected in plasma. We observed lower plasma levels in acute HF patients, while chronic patients did not show changes. In fact, during an acute phase of HF, plasma FAP levels decrease regardless of the etiology, functional class, echocardiographic parameters, and other clinical variables. This finding contributes to progress in the knowledge of cardiac fibrosis and raises questions about the role of FAP in the diagnosis of acute HF.

### Supplementary Information

**Additional file 1: Table S1.** Non-statistically significant correlations of *FAP* with genes of collagens, ECM structural constituents and TGF-β signaling pathway*.* **Table S2.** Non-statistically significant correlations of *FAP* with profibrotic and antifibrotic microRNAs. **Figure S1.** Differential expression levels of downstream molecules of TGF-β signaling in HF patients.

## Data Availability

Data discussed in this publication have been deposited in NCBI’s Gene Expression Omnibus (GEO) [52] and are accessible through GEO Series accession number GSE55296 (http://www.ncbi.nlm.nih.gov/geo/query/acc.cgi?acc=GSE55296).

## Abbreviations

BMI: Body mass index
ECM: Extracellular matrix
FAP: Fibroblast activation protein-α
HF: Heart failure
NYHA: New York Heart Association
ncRNA: Noncoding RNA
TGF-β: Transforming growth factor-β
