# Supplementary material for: Plasma fibroblast activation protein is decreased in acute heart failure despite cardiac tissue upregulation
Source: J Transl Med. 2024 Feb 1;22:124. doi: 10.1186/s12967-024-04900-w (PMC10832198; doi:10.1186/s12967-024-04900-w)
Supplement: Supplementary file 1 — Additional file 1: Table S1. Non-statistically significant correlations of FAP with genes of collagens, ECM structural constituents and TGF-β signaling pathway. Table S2. Non-statistically significant correlations of FAP with profibrotic and antifibrotic microRNAs. Figure S1. Differential expression levels of downstream molecules of TGF-β signaling in HF patients. [file 12967_2024_4900_MOESM1_ESM.docx]

**Additional file 1**

**Table S1.** Non-statistically significant correlations of *FAP* with genes of collagens, ECM structural constituents and TGF-β signaling pathway*.*

| **Gene** | **Name** | **Description** | **Correlation with *FAP*** | |
| --- | --- | --- | --- | --- |
|  |  |  | **r** | ***p*** |
| *COL1A1* | Collagen type I alpha 1 chain | Fibrillar collagen | 0.294 | 0.092 |
| *COL4A1* | Collagen type IV alpha 1 chain | Nonfibrillar collagen | 0.321 | 0.064 |
| *COL4A5* | Collagen type IV alpha 5 chain |  | 0.056 | 0.750 |
| *MMP2* | Matrix metallopeptidase 2 | ECM structural constituents | 0.104 | 0.553 |
| *ELN* | Elastin |  | 0.299 | 0.081 |
| *LOXL1* | Lysyl oxidase like 1 | LOX family | 0.112 | 0.521 |
| *TGFB3* | Transforming growth factor beta 3 | TGF-β signaling pathway | 0.295 | 0.091 |
| *TGFBR2* | Transforming growth factor beta receptor 2 |  | -0.077 | 0.661 |
| *SMAD3* | SMAD family member 3 |  | -0.309 | 0.071 |
| *SMAD6* | SMAD family member 6 |  | 0.192 | 0.268 |
| *MAPK8* | Mitogen-activated protein kinase 8 |  | -0.184 | 0.291 |
| *STAT3* | Signal transducer and activator of transcription 3 |  | -0.126 | 0.469 |

ECM:extracellular matrix

**Table S2.** Non-statistically significant correlations of *FAP* with profibrotic and antifibrotic microRNAs.

| **MicroRNA** | **Description** | **Correlation with *FAP*** | |
| --- | --- | --- | --- |
|  |  | **r** | ***p*** |
| MiR-155-5p | Profibrotic microRNAs | 0.317 | 0.131 |
| MiR-483-5p |  | -0.035 | 0.872 |
| MiR-1-3p | Antifibrotic microRNAs | -0.177 | 0.409 |
| MiR-133a-3p |  | -0.109 | 0.613 |
| MiR-19b-3p |  | -0.171 | 0.425 |
| MiR-29c-3p |  | -0.137 | 0.522 |
| MiR-590-3p |  | -0.322 | 0.125 |


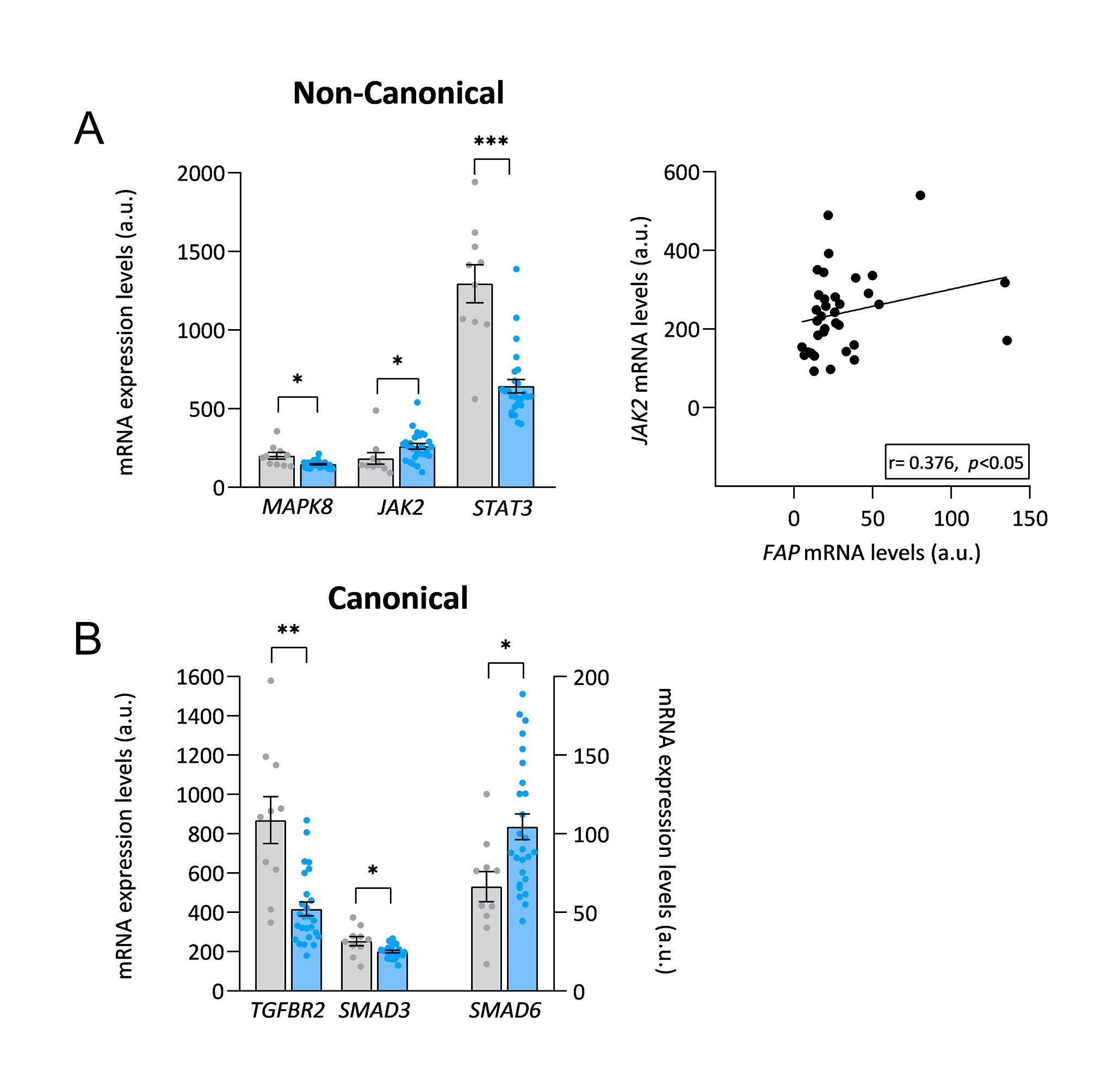


**Figure S1.** **Differential expression levels of downstream molecules of TGF-β signaling in HF patients.** (**A**) Relative mRNA expression levels of members of non-canonical pathway. Correlation of *FAP* mRNA levels with *JAK2* mRNA levels. (**B**) Relative mRNA expression levels of members of the canonical pathway. Figures reflect the relative expression (in arbitrary units) extracted from the mRNA-seq analysis for both patients and controls to provide an idea of the average expression of each molecule. A.u., arbitrary units. Correlations were determined using Spearman’s correlation coefficient. Control subjects (gray), HF patients (light blue).* *p*<0.05, ** *p*<0.01, *** *p*<0.001. HF - heart failure; *JAK2* - janus kinase 2; *MAPK8 -* mitogen-activated protein kinase 8; *SMAD3 -* SMAD family member 3; *SMAD6* - SMAD family member 6; *STAT3* - signal transducer and activator of transcription 3; *TGFBR2* - transforming growth factor beta receptor 2.
